# Primary urothelial carcinoma of the prostate: report of two cases and review of the literature

**DOI:** 10.3332/ecancer.2025.2022

**Published:** 2025-10-22

**Authors:** Panzardi Nicolás, Fernández-Alberti Joaquin, Schinoni Juan Pablo, Ares Jorge Hugo, Iotti Alejandro, Featherston Marcelo

**Affiliations:** 1 Department of Urology, British Hospital of Buenos Aires, Buenos Aires 1280, Argentina; 2 Department of Anatomic Pathology, British Hospital of Buenos Aires, Buenos Aires 1280, Argentina

**Keywords:** urothelial carcinoma, prostate carcinoma, transitional cell, prostatic urethra, prostate cancer

## Abstract

Transitional cell carcinoma of the prostatic urethra was first described and reported in 1963. Primary urothelial carcinoma of the prostate is an extremely rare disease, often misdiagnosed with other entities, particularly with urothelial carcinoma of the bladder involving the prostatic tissue. There are only dozens of cases of primary urothelial carcinoma in the prostate reported in the literature. The prognosis after diagnosis is reported to be poor and there is no consensus among experts concerning its proper treatment and follow-up. Herein, we report two cases of patients diagnosed with this entity with a median follow-up of 14 months.

## Introduction

Urothelial carcinoma can occur either in the upper urinary tract or in the lower urinary tract (bladder and urethra). However, primary urothelial carcinoma in the prostate (PUCP) gland is extremely rare [3].

According to the 2020 Global Cancer Statistics, prostate cancer is the second most common malignancy in men, only after lung cancer. Over 90% of these prostatic tumours present adenocarcinoma as a histological subtype. Besides adenocarcinoma, prostatic tumours can grow from stromal tissue, and urothelial carcinoma can grow from the prostatic ducts or the mucosae from the prostatic transitional urothelium [1].

## Objective

To report two cases of patients diagnosed with PUCP and to perform a review of the available literature to clarify the diagnostic pitfalls and treatment options in this particular entity.

## Case presentation: patient 1

We present the case of a 77-year-old male patient with a clinical history of high blood pressure, diabetes and urothelial bladder carcinoma (diagnosed and resected in 2003). Pathological examination of the lesion confirmed that the low-grade urothelial carcinoma was non-muscle invasive. The patient also underwent transurethral resection of the prostate (TURP) due to symptomatic benign hyperplasia in April, 2023, for a prostatic weight of 59 g. His serum prostate-specific antigen (PSA) level was 3.4 ng/mL at the moment.

One month following the TURP, the patient developed symptoms of low-urinary tract obstruction again. Flexible cystoscopy was performed in the patient, showing synechiae in the surgical lodge. In June 2023, a new TURP was performed to resolve his urinary obstruction symptoms. The pathological findings were consistent with fibroglandular hyperplasia plus focal, isolated involvement of the intraprostatic canaliculus by high-grade urothelial carcinoma. Absence of infiltration of the prostatic stroma.

After a thorough discussion of the case, the multidisciplinary consensus opinion was to initiate induction with intravesical Calmette-Guerin Bacillus (BCG) and then to perform a magnetic resonance imaging (MRI) scan. No pathological findings were observed in the resonance image. It was then decided to perform treatment with BCG in maintenance doses. At this time, 1 month after the MRI was performed, the patient presented with macroscopic hematuria. Cystoscopy was performed at the time, observing two polypoid lesions of 10 mm each, located in the prostatic urethra associated with non-pathological bladder mucosae.

After further discussion, TURP was performed. Histopathological examination of the specimen revealed that the features of the prostate lesions were consistent with high-grade urothelial carcinoma of the prostate. Isolated and focal compromise of the tissue.

**Figure 1.**
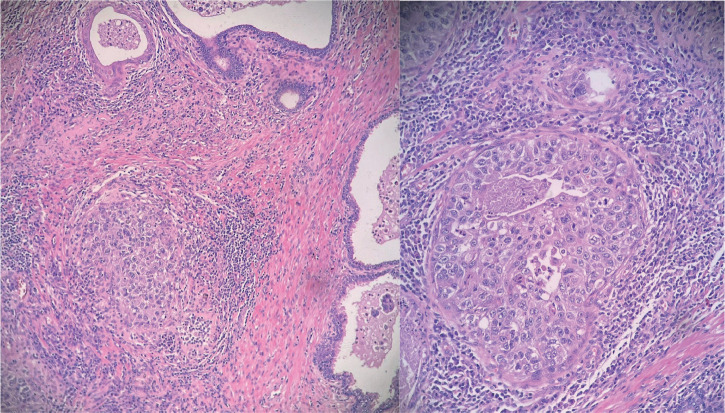
Hematoxylin and eosin stained samples (magnification x100 and x400). Tumour cells exhibit a characteristic 'umbrella'-like architectural pattern, without invading the prostatic stroma. They show abundant, clear cytoplasm, with marked nuclear atypia.

With the pathological result, the patient was reconsidered once again upon multidisciplinary consensus. The final decision was to perform a radical prostatectomy in May 2024. The final diagnosis was compatible with high-grade urothelial carcinoma of the prostate, compromising the intraprostatic ducts and the right seminal vesicle, with free margins. Overall prostatic compromise of about 5%. The absence of extraprostatic extension and vascular or perineural involvement was demonstrated (Figure 1).

At 6 months of follow-up, transurethral resection of the bladder was performed due to a polypoid lesion found by cystoscopy. The pathological findings were compatible with low-grade urothelial carcinoma Ta.

No further treatment was administered; the patient presented no pathological findings in the cystoscopic examination, and his urinary cytology was negative for neoplastic cells.

## Case presentation: patient 2

We present the case of a 77-year-old male patient with a clinical history of tabagism (50 PY), high blood pressure, diabetes mellitus and urothelial bladder carcinoma, which was diagnosed and resected in 2016 for the very first time, with multiple resections between 2016 and 2019. Pathological examination of the lesion demonstrated a high-grade urothelial carcinoma that was non-muscle invasive. In the follow-up, the patient developed infravesical obstruction symptoms. Cystoscopy revealed a sclerotic bladder neck, with no observable mucosal lesión in the bladder. He then underwent TURP in order to improve his urinary symptoms in February 2022. Pathological examination of the samples revealed intracanalicular compromise of the prostatic tissue by high-grade urothelial carcinoma, with the presence of necrotic areas. Treatment with BCG was performed at that time, and then the patient underwent a new cystoscopy in which no lesions were visible. A new TURP was decided, and pathology informed no oncologic findings in the samples, including the resection of the surgical lodge. Cistoscopy and urine cytology (PAP) were negative during his follow-up. His serum PSA level was 3.4 ng/mL at the time of the report of his case.

**Figure 2.**
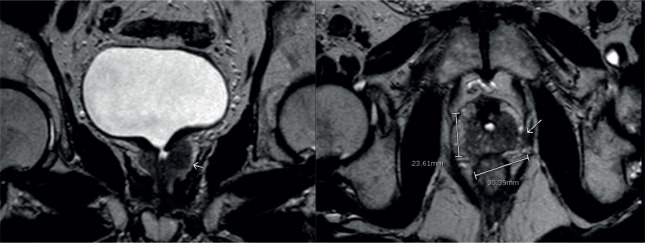
MRI shows an infiltrative tissue located in the left prostatic lobe. It is hypointense in the T2 sequence and enhances after endovenous contrast. It measures 30.3 × 23.6 mm.

In May, 2023, the patient developed obstruction symptoms once again. Urine cytology was repeated, which was positive for neoplastic cells. He had a computed tomography (CT) scan that showed no significant lesions. He had a MRI which showed a poorly delimited tissue, hypointense in T2 sequence, which enhanced after contrast administration, surrounding the bladder neck and the prostatic urethra, showing signs of possible extra-prostatic compromise (Figure 2).

A positron emission tomography (PET)/CT image was obtained, showing hypercaptation of the left prostatic lobe, without any other sites of tumour localisation.

His rectal digital examination revealed a solid lesion located in the left prostatic lobe. A transrectal biopsy of this lesion was performed, and the pathological examination features of the biopsy specimen were consistent with infiltration of urothelial high-grade carcinoma. The GATA-3 marker was found to be positive in the sample. NKX3.1 and CK 7 markers were negative in the studied tissue.

The case of the patient was discussed among urology experts, oncologists and pathologists. The multidisciplinary consensus was to offer the patient surgical resection of the tumour.

Laparoscopic radical prostatectomy with pelvic lymph node dissection was performed in November 2023. Pathological examination of the specimen revealed high-grade urothelial carcinoma that had originated in the prostatic canaliculus, invading the left prostatic lobe, compromising 15% of the prostate. Perineural and lymphatic vascular invasion was observed. The resection margins were tumour-free. His seminal vesicles showed no tissue invasion by the carcinoma. None of the six lymph nodes resected was positive for neoplasm.

At the time of the case report, the patient was at 16 months of his follow-up. He presented with neither hemathuria nor other symptoms. He underwent cystoscopy, which showed no signs of recurrence. His urine cytology was negative for cancerous cells. His serum PSA levels were <0.1 ng/mL.

## Discussion

Patients suffering from this entity may lack symptoms or present with non-specific lower urinary tract symptoms, such as hematuria, urinary frequency, nocturia, lower abdominal pain or dysuria. Gross hematuria has also been described as the clinical manifestation of this disease [2]. Other uncommon symptoms have been reported, including sustained fever, rectal bleeding or even a palpable abdominal wall nodule (which was diagnosed as a metastatic lesion) [5, 6].

These symptoms described above are similar to those presented by patients suffering from prostatic benign hyperplasia or prostatic adenocarcinoma, which makes it challenging to differentiate one entity from another by clinical findings. Although hard prostatic nodules in the digital examination can be evidenced in some patients, this does not allow the physician to distinguish between PUCP and prostatic adenocarcinoma.

The initial evaluation consisted of abdominal ultrasonography and cystoscopy. MRI or CT is indicated in cases where previous studies are inconclusive or do not provide evidence of pathological findings. MRI presents higher sensitivity in diagnosing intraprostatic solid lesions and the presence of extraprostatic dissemination. In the published literature, there are studies reporting up to 88.9% of PUCP that were evidenced with MRI in the cohort [2].

Fluorodeoxyglucose PET has also shown high sensitivity in showing not only PUCP but also its distant metastases. The lack of specificity of the radiological features makes it difficult to distinguish PUCP from other types of prostate cancer [7].

In addition, urinary cytology is indicated in patients with hematuria. It presents high specificity but low sensibility in diagnosing urothelial carcinoma, due to the fact that it diagnoses high-grade carcinoma cells.

Serum PSA is mandatory in these patients. PUCP does not show high PSA levels, but it can be high in patients with prostate adenocarcinoma or in those presenting benign prostatic hyperplasia.

The confirmatory diagnosis consists of histological and immunohistochemical markers in the tissue samples. These biopsies may be obtained from transurethral resection or by transrectal ultrasonography-guided biopsy. The coexpression of CK7 and CK20 markers, CK34βE12, GATA-3 and p63 presence are the key for arriving at a correct diagnosis of PUCP. Also, prostatic markers such as NKX3.1 should be negative in these samples and PSA is not elevated in patients with urothelial carcinoma [8].

Sensitivity for diagnosing prostate adenocarcinoma with PSA level and NKX3.1 are nearly 100% and 88.3%, respectively, whereas CK34βE12, p63 and GATA3 are not usually present in prostatic adenocarcinoma (1.8%, 0% and 0%, respectively, in prostatic adenocarcinoma tissue) [8].

The diagnostic rate is about 40% for all the samples obtained by transrectal needle biopsy, while transurethral prostatic resection with biopsy has a diagnostic rate of around 90% [4].

PUCP is usually diagnosed as a high-grade urothelial carcinoma. At the initial stages of diagnosis, it usually presents as a T3-T4 tumour and is mostly associated with a poor prognosis [2, 3].

Approximately, 50% of patients diagnosed with PUCP present with stage T3 or T4 disease, and approximately 20% of these patients have distant metastasis at the time of diagnosis. These tend to be found within the bone, lung and liver because of their high degree of malignancy and strong invasiveness [2].

The PUCP does not have a consensus concerning its optimal management. Treatment principles depend on the fact that the histopathological diagnosis is a urothelial carcinoma. Therefore, PUCP is usually treated as a bladder urothelial carcinoma. Some authors consider transurethral resection and administration of the Calmette-Guerrin vaccine the first treatment method for all patients presenting with non-stromal-invasive PUCP located in the prostatic urethra. For those tumours invading the prostatic stroma, radical surgery is often recommended (cystoprostatectomy or radical prostatectomy) [9].

Radiotherapy and adjuvant therapies are also useful in these cases. The standard systemic therapy comprises radiotherapy associated with gemcitabine plus cisplatin. This approach is quite different from the standard treatment for prostatic adenocarcinoma, but it is similar to the one indicated for urothelial bladder carcinoma [2].

Surgical options vary from radical surgery (cystoprostatectomy or radical prostatectomy alone), radiotherapy or adjuvant therapy.

As a non-hormone-dependent tumour, it is known that androgen-deprivation therapy is not useful in this particular carcinoma.

The prognosis after diagnosis is often poor in this pathology. The 10-year overall survival (OS) rate of patients with typical prostatic adenocarcinoma is over 70% according to the worldwide literature. However, in the case of PUCP, the median OS has been reported to range from 4.6 to 42 months [10].

Unlike the bladder urothelium, the prostatic duct urothelium lacks a layer of lamina propria, thus facilitating penetration of the basal membrane and invasion of the prostatic stroma by tumour, which leads to worse results in OS.

## Conclusion

PUCP gland is a rare and aggressive disease with a low life expectancy after diagnosis. Even though it arises in the prostatic ducts, its treatment and prognosis differ from those of adenocarcinoma of the prostate. It should be suspected in patients presenting with lower urinary tract symptoms, in particular hematuria or dysuria, usually associated with normal serum PSA levels. MRI is the most useful imaging technique, and histological biopsy should be performed by transurethral resection whenever possible. Typical immunohistochemistry findings were positive for GATA-3 and P63 and negative for PSA and NKX3.1. TURP associated with BCG treatment, radical cystectomy or cystoprostatectomy, chemotherapy with gemcitabine and cisplatin and radiotherapy can be effective. Large, well-designed prospective studies should be conducted in the future in order to obtain further information about this rare disease.

## Conflicts of interest/funding

We state that we have neither funding nor conflicts of interest to declare of any type regarding the present manuscript. Also, both patients have given their informed consent to report the cases.
